# The First American Symphyseotomy

**Published:** 1894-04

**Authors:** 


### THE FIRST AMERICAN SYMPHYSEOTOMY.

It appears that Dr. William T. Coggin, now of Athens, Ga., was the first to make a symphyseotomy in this country. Up to last October it was thought that this distinction belonged to Professor Jewett, of Brooklyn, who operated September 30, 1892.

However, on March 12, 1892, Dr. Coggin, then living at Keener, Ala., operated with entire success both to mother and child. His patient was a primipara, aged twenty-three, who had been fifteen hours in labor when he was called. Failing to deliver on account of contracted pelvis, he opened the symphysis and extracted the child. The pubic bones separated two and three-quarter inches. They readily reunited under proper restraining apparatus.

We are indebted for this information to a late article by Dr. R. P. Harris, of Philadelphia, in the *Medical and Surgical Reporter*.

